# Supplementary material for: HIVprotI: an integrated web based platform for prediction and design of HIV proteins inhibitors
Source: J Cheminform. 2018 Mar 9;10:12. doi: 10.1186/s13321-018-0266-y (PMC5845081; doi:10.1186/s13321-018-0266-y)
Supplement: Supplementary file 2 — Additional file 2. Source code of HIVProtI web server. [file 13321_2018_266_MOESM2_ESM.zip › HIVprotI_Source-code/hivprotI/weka-3-6-6/doc/weka/filters/supervised/attribute/ClassOrder.html]

ClassOrder


---


|  |  |  |
| --- | --- | --- |
| **Overview** \| **Package** \| **Class** \| **Tree** \| **Deprecated** \| **Index** \| **Help** \| **Weka's home** | |  |
| **PREV CLASS**   **NEXT CLASS** | **FRAMES**    **NO FRAMES**     **All Classes** |  |
| SUMMARY: NESTED \| FIELD \| CONSTR \| METHOD | DETAIL: FIELD \| CONSTR \| METHOD |  |


---


## weka.filters.supervised.attribute Class ClassOrder

```
java.lang.Object
  weka.filters.Filter
      weka.filters.supervised.attribute.ClassOrder
```

**All Implemented Interfaces:**: java.io.Serializable, CapabilitiesHandler, OptionHandler, RevisionHandler, SupervisedFilter

---

``` public class ClassOrder extends Filter implements SupervisedFilter, OptionHandler ```

Changes the order of the classes so that the class values are no longer of in the order specified in the header. The values will be in the order specified by the user -- it could be either in ascending/descending order by the class frequency or in random order. Note that this filter currently does not change the header, only the class values of the instances, so there is not much point in using it in conjunction with the FilteredClassifier. The value can also be converted back using 'originalValue(double value)' procedure.


Valid options are:

```
 -R <seed>
  Specify the seed of randomization
  used to randomize the class
  order (default: 1)
```

```
 -C <order>
  Specify the class order to be
  sorted, could be 0: ascending
  1: descending and 2: random.(default: 0)
```

**Version:**
:   $Revision: 5541 $

**Author:**
:   Xin Xu (xx5@cs.waikato.ac.nz), Eibe Frank (eibe@cs.waikato.ac.nz)

**See Also:**: Serialized Form

---

| **Field Summary** | |
| --- | --- |
| `static int` | `FREQ_ASCEND`             The class values are sorted in ascending order based on their frequencies |
| `static int` | `FREQ_DESCEND`             The class values are sorted in descending order based on their frequencies |
| `static int` | `RANDOM`             The class values are sorted in random order |


**Constructor Summary**

`ClassOrder()`


| **Method Summary** | |
| --- | --- |
| `boolean` | `batchFinished()`             Signify that this batch of input to the filter is finished. |
| `java.lang.String` | `classOrderTipText()`             Returns the tip text for this property |
| `double[]` | `distributionsByOriginalIndex(double[] before)`             Convert the given class distribution back to the distributions with the original internal class index |
| `Capabilities` | `getCapabilities()`             Returns the Capabilities of this filter. |
| `double[]` | `getClassCounts()`             Get the class distribution of the sorted class values. |
| `int` | `getClassOrder()`             Get the wanted class order |
| `java.lang.String[]` | `getOptions()`             Gets the current settings of the filter. |
| `java.lang.String` | `getRevision()`             Returns the revision string. |
| `long` | `getSeed()`             Get the current randomization seed |
| `java.lang.String` | `globalInfo()`             Returns a string describing this filter |
| `boolean` | `input(Instance instance)`             Input an instance for filtering. |
| `java.util.Enumeration` | `listOptions()`             Returns an enumeration describing the available options. |
| `static void` | `main(java.lang.String[] argv)`             Main method for testing this class. |
| `double` | `originalValue(double value)`             Return the original internal class value given the randomized class value, i.e. |
| `java.lang.String` | `seedTipText()`             Returns the tip text for this property |
| `void` | `setClassOrder(int order)`             Set the wanted class order |
| `boolean` | `setInputFormat(Instances instanceInfo)`             Sets the format of the input instances. |
| `void` | `setOptions(java.lang.String[] options)`             Parses a given list of options. |
| `void` | `setSeed(long seed)`             Set randomization seed |

**Methods inherited from class weka.filters.Filter**

`batchFilterFile, filterFile, getCapabilities, getOutputFormat, isFirstBatchDone, isNewBatch, isOutputFormatDefined, makeCopies, makeCopy, numPendingOutput, output, outputPeek, toString, useFilter, wekaStaticWrapper`

**Methods inherited from class java.lang.Object**

`equals, getClass, hashCode, notify, notifyAll, wait, wait, wait`

**Field Detail**

### FREQ\_ASCEND

```
public static final int FREQ_ASCEND
```

:   The class values are sorted in ascending order based on their frequencies

    **See Also:**: Constant Field Values

---


### FREQ\_DESCEND

```
public static final int FREQ_DESCEND
```

:   The class values are sorted in descending order based on their frequencies

    **See Also:**: Constant Field Values

---


### RANDOM

```
public static final int RANDOM
```

:   The class values are sorted in random order

    **See Also:**: Constant Field Values


**Constructor Detail**

### ClassOrder

```
public ClassOrder()
```


**Method Detail**

### globalInfo

```
public java.lang.String globalInfo()
```

:   Returns a string describing this filter

    :   **Returns:**: a description of the filter suitable for displaying in the explorer/experimenter gui

---


### listOptions

```
public java.util.Enumeration listOptions()
```

:   Returns an enumeration describing the available options.

    :   **Specified by:**: `listOptions` in interface `OptionHandler`
    :   **Returns:**: an enumeration of all the available options.

---


### setOptions

```
public void setOptions(java.lang.String[] options)
                throws java.lang.Exception
```

:   Parses a given list of options.


    Valid options are:

    ```
     -R <seed>
      Specify the seed of randomization
      used to randomize the class
      order (default: 1)
    ```

    ```
     -C <order>
      Specify the class order to be
      sorted, could be 0: ascending
      1: descending and 2: random.(default: 0)
    ```

    :   **Specified by:**: `setOptions` in interface `OptionHandler`
    :   **Parameters:**: `options` - the list of options as an array of strings **Throws:**: `java.lang.Exception` - if an option is not supported

---


### getOptions

```
public java.lang.String[] getOptions()
```

:   Gets the current settings of the filter.

    :   **Specified by:**: `getOptions` in interface `OptionHandler`
    :   **Returns:**: an array of strings suitable for passing to setOptions

---


### seedTipText

```
public java.lang.String seedTipText()
```

:   Returns the tip text for this property

    :   **Returns:**: tip text for this property suitable for displaying in the explorer/experimenter gui

---


### getSeed

```
public long getSeed()
```

:   Get the current randomization seed

    :   **Returns:**: a seed

---


### setSeed

```
public void setSeed(long seed)
```

:   Set randomization seed

    :   **Parameters:**: `seed` - the set seed

---


### classOrderTipText

```
public java.lang.String classOrderTipText()
```

:   Returns the tip text for this property

    :   **Returns:**: tip text for this property suitable for displaying in the explorer/experimenter gui

---


### getClassOrder

```
public int getClassOrder()
```

:   Get the wanted class order

    :   **Returns:**: class order

---


### setClassOrder

```
public void setClassOrder(int order)
```

:   Set the wanted class order

    :   **Parameters:**: `order` - the class order

---


### getCapabilities

```
public Capabilities getCapabilities()
```

:   Returns the Capabilities of this filter.

    :   **Specified by:**: `getCapabilities` in interface `CapabilitiesHandler` **Overrides:**: `getCapabilities` in class `Filter`
    :   **Returns:**: the capabilities of this object **See Also:**: `Capabilities`

---


### setInputFormat

```
public boolean setInputFormat(Instances instanceInfo)
                       throws java.lang.Exception
```

:   Sets the format of the input instances.

    :   **Overrides:**: `setInputFormat` in class `Filter`
    :   **Parameters:**: `instanceInfo` - an Instances object containing the input instance structure (any instances contained in the object are ignored - only the structure is required). **Returns:**: true if the outputFormat may be collected immediately **Throws:**: `java.lang.Exception` - if no class index set or class not nominal

---


### input

```
public boolean input(Instance instance)
```

:   Input an instance for filtering. Ordinarily the instance is processed
    and made available for output immediately. Some filters require all
    instances be read before producing output.

    :   **Overrides:**: `input` in class `Filter`
    :   **Parameters:**: `instance` - the input instance **Returns:**: true if the filtered instance may now be collected with output(). **Throws:**: `java.lang.IllegalStateException` - if no input format has been defined.

---


### batchFinished

```
public boolean batchFinished()
                      throws java.lang.Exception
```

:   Signify that this batch of input to the filter is finished. If
    the filter requires all instances prior to filtering, output()
    may now be called to retrieve the filtered instances. Any
    subsequent instances filtered should be filtered based on setting
    obtained from the first batch (unless the inputFormat has been
    re-assigned or new options have been set). This implementation
    sorts the class values and provide class counts in the output format

    :   **Overrides:**: `batchFinished` in class `Filter`
    :   **Returns:**: true if there are instances pending output **Throws:**: `java.lang.IllegalStateException` - if no input structure has been defined,: `java.lang.Exception` - if there was a problem finishing the batch.

---


### getClassCounts

```
public double[] getClassCounts()
```

:   Get the class distribution of the sorted class values. If class is numeric
    it returns null

    :   **Returns:**: the class counts

---


### distributionsByOriginalIndex

```
public double[] distributionsByOriginalIndex(double[] before)
```

:   Convert the given class distribution back to the distributions
    with the original internal class index

    :   **Parameters:**: `before` - the given class distribution **Returns:**: the distribution converted back

---


### originalValue

```
public double originalValue(double value)
                     throws java.lang.Exception
```

:   Return the original internal class value given the randomized
    class value, i.e. the string presentations of the two indices
    are the same. It's useful when the filter is used within a classifier
    so that the filtering procedure should be transparent to the
    evaluation

    :   **Parameters:**: `value` - the given value **Returns:**: the original internal value, -1 if not found **Throws:**: `java.lang.Exception` - if the coverter table is not set yet

---


### getRevision

```
public java.lang.String getRevision()
```

:   Returns the revision string.

    :   **Specified by:**: `getRevision` in interface `RevisionHandler` **Overrides:**: `getRevision` in class `Filter`
    :   **Returns:**: the revision

---


### main

```
public static void main(java.lang.String[] argv)
```

:   Main method for testing this class.

    :   **Parameters:**: `argv` - should contain arguments to the filter: use -h for help


---


|  |  |  |
| --- | --- | --- |
| **Overview** \| **Package** \| **Class** \| **Tree** \| **Deprecated** \| **Index** \| **Help** \| **Weka's home** | |  |
| **PREV CLASS**   **NEXT CLASS** | **FRAMES**    **NO FRAMES**     **All Classes** |  |
| SUMMARY: NESTED \| FIELD \| CONSTR \| METHOD | DETAIL: FIELD \| CONSTR \| METHOD |  |


---
